# Effects of the different periods and magnitude of COVID‐19 infection spread on cancer operations: Interrupted time series analysis of medical claims data

**DOI:** 10.1002/cam4.5259

**Published:** 2022-09-20

**Authors:** Natsue Kashiwagura, Fuyuhiko Motoi, Upul Cooray, Ryu Fukase, Yukiko Katayama, Ken Osaka, Masayasu Murakami, Takaaki Ikeda

**Affiliations:** ^1^ Department of Health Policy Science, Graduate School of Medical Science Yamagata University Yamagata Japan; ^2^ Yamagata City Hospital Saiseikan Yamagata Japan; ^3^ Department of Surgery I, Graduate School of Medical Science Yamagata University Yamagata Japan; ^4^ Department of International and Community Oral Health Tohoku University Graduate School of Dentistry Sendai Japan; ^5^ Sakakibara Heart Institute Fuchu Japan

**Keywords:** malignant neoplasm, medical care system, SARS‐CoV‐2, scheduled treatment, severe acute respiratory syndrome coronavirus‐2

## Abstract

**Background:**

No clear evidence exists regarding the effects of the different periods and magnitude of spread of the COVID‐19 infection on cancer treatments. This study investigated the effects of the different periods and magnitude of COVID‐19 infection spread on in‐hospital cancer operations.

**Methods:**

Medical claims data from 17 hospitals where in‐hospital operations for patients with malignant neoplasms were performed between 1 April 2017 and 31 March 2021 in Yamagata were extracted and analyzed. The critical time points as exposure used to evaluate the impact of different COVID‐19 infection spread periods on cancer operations were (1) April 2020 (emergency declaration introduced by the government) and (2) December 2020 (the second wave). From April to November 2020 and December 2020 to March 21, the number of confirmed COVID‐19 cases was 130 and 840, respectively. The 17 hospitals were classified into intervention or control groups based on whether in‐hospital treatments for patients with COVID‐19 were provided.

**Results:**

The interrupted time series analysis reported that the difference in the trend of pre‐COVID‐19 and postsecond wave (March 2020 to December 2020) periods was statistically significant between groups, with 50.67 fewer operations (95% confidence interval [CI] = 12.19–89.15) performed per month in the intervention group compared with the control group. Moreover, the immediate change in the number of operations in April 2020 (beginning of the first wave) was statistically significant between groups, with 80.14 operations (95% CI = 39.62–120.67) less immediately after the first wave in the intervention group compared with the control group.

**Conclusion:**

Our findings suggest that a statement of emergency by the government and the COVID‐19 infection spread are both associated with the number of cancer operations performed in the Yamagata prefecture during the COVID‐19 pandemic.

## Graphical abstract

This retrospective observational study used medical claims data in Japan to examine the effects of different COVID‐19 periods and magnitudes of its spread on in‐hospital cancer operations. We confirmed that cancer operations were significantly reduced in hospitals where in‐hospital treatments for patients with COVID‐19 were provided during the COVID‐19 pandemic. Our findings suggest that a statement of emergency by the government as well as the spread of COVID‐19 both have an influence on the number of cancer operations performed in Yamagata prefecture during the COVID‐19 era.

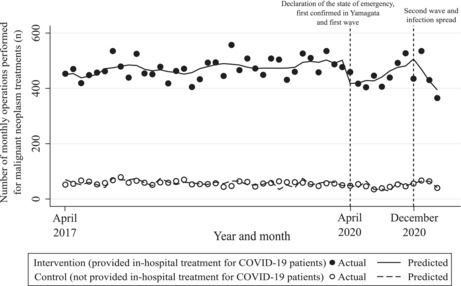

## 1 INTRODUCTION

The global outbreak of the novel coronavirus, known as severe acute respiratory syndrome coronavirus‐2 (SARS‐CoV‐2), has caused devastating effects on healthcare systems worldwide.^1^, ^2^, ^3^, ^4^, ^5^, ^6^, ^7^, ^8^, ^9^, ^10^ Several medical resources, such as intensive care units and medical professionals, had been diverted toward coronavirus disease‐2019 (COVID‐19) treatment and prevention.^11^ Consequently, several scheduled treatments for various diseases, including in‐hospital operations for patients with cancer, were disrupted.^6^, ^7^, ^8^, ^9^, ^10^

To date, numerous studies have reported on cancer treatment disruption due to the COVID‐19 pandemic. However, they were based on a small number of patients,^8^ affected by the recall bias because of self‐reported data,^12^ or did not investigate other possible factors of treatment delays (e.g. seasonality) due to the absence of a control group.^6^, ^7^ Furthermore, the abovementioned studies were conducted during the early COVID‐19 pandemic period and in countries where COVID‐19 rapidly spread following the first confirmed case.^13^ Therefore, the effects of different periods and the magnitude of COVID‐19 spread on cancer treatments had not been clarified.

The present study applied a “natural experiment design”^14^ that enabled the distinction between the different COVID‐19 periods, including the first confirmed infection in the local community and the period during which the infection spread. This retrospective observational study was conducted using medical claims data collected from multiple hospitals in Yamagata prefecture, one of the prefectures (states) of Japan, to investigate the effect of different COVID‐19 periods and magnitudes of spread on scheduled in‐hospital operations for patients with cancer.

## 2 MATERIALS AND METHODS

### 2.1 Data source and study population

The diagnosis procedure combination (DPC) data were obtained from all hospitals in Yamagata prefecture. The hospitals where in‐hospital operations for patients with malignant neoplasms (ICD‐10 codes C00–C97) were recorded were eligible for the study (*N* = 28). The DPC system is a scheme for bundled payment for acute‐phase inpatient hospital for profiling healthcare services, which included clinical information of each patient, such as their age, sex, primary diagnosis (by ICD‐10 codes), comorbidities at admission, emergency or scheduled hospitalization, concomitant chemotherapy, and cancer recurrence. The details regarding the DPC system have been described elsewhere.^15^, ^16^ The data regarding discharged patients from 1 April 2017 to 31 March 2021 were analyzed in this study. We excluded 11 hospitals with <100 cases during the observation periods, and the remaining 17 hospitals were included in the main analysis. Overall, 97% of patients in Yamagata receive medical care services within Yamagata,^17^ and the DPC data cover 98% of all operations in the Yamagata prefecture.^18^ Therefore, the data in the present study are highly comprehensive in terms of Yamagata.^18^

### 2.2 Exposure

**FIGURE 1:**
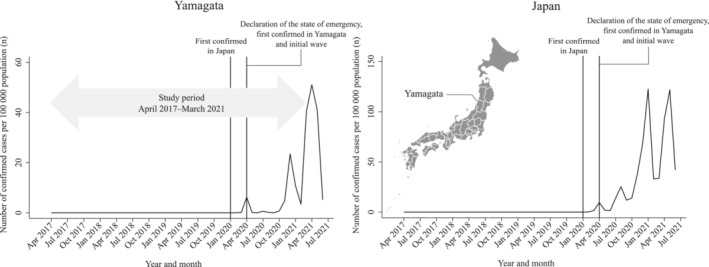
Number of confirmed COVID‐19 cases per 100,000 population according to Japan and Yamagata.

Figure 1 summarizes the number of confirmed COVID‐19 cases in Japan and Yamagata prefecture. Yamagata prefecture is located in the northern district of Japan and has a population of approximately 1 million residents. In Japan, the first COVID‐19 patient was confirmed on 16 January 2020.^19^ Meanwhile, in Yamagata, the first COVID‐19 patient was identified on 31 March 2020, and by the end of May 2020, a total of 69 patients were confirmed to be positive for COVID‐19 infection (the first wave).^20^ At the same time, on 16 April 2020, the government issued a nationwide state of emergency, requesting that residents refrain from nonessential activities and maintain physical distance.^19^ The state of emergency throughout Japan was removed on 31 May 2020, and the declaration in Yamagata has not been issued since then.^19^ Thus, the first wave of infections in Yamagata and the government's declaration of a state of emergency occurred at approximately the same time, and no rapid spread of infection was confirmed in the first wave. No cases were confirmed in June 2020. After October 2020, the number of confirmed COVID‐19 cases gradually increased and reached its peak in December 2020, when 253 infected people were confirmed, and the number of infected people expanded rapidly in Yamagata (the second wave).^20^

We used the following critical time points to evaluate the impact of the different periods and magnitude of COVID‐19 infection spread on scheduled cancer operations: (1) April 2020 (the first COVID‐19 case confirmed in Yamagata; the initial wave of COVID‐19 cases, and the nationwide state of emergency was declared) and (2) December 2020 (the peak of the second wave of confirmed COVID‐19 cases) (Figure 1). Because the first COVID‐19 patient in Yamagata was confirmed on 31 March 2020, we considered April 2020 as the first time point.

### 2.3 Intervention and control groups

In Japan, medical systems have been established on a prefectural basis, and Yamagata prefecture designated hospitals to provide inpatient treatment for patients with COVID‐19. Consequently, these hospitals were prioritizing restrictions on accepting inpatients to establish a medical system that includes infection prevention measures and securing hospital beds.

The 17 hospitals were first classified into two groups based on whether in‐hospital treatments for patients with COVID‐19 were provided. To ensure that patients with COVID‐19 were not admitted to a hospital not designated by the local government, we used the ICD‐10 code of U‐071. Nine hospitals (the number of hospital beds in the hospitals was ≥400) provided in‐hospital treatments for patients with COVID‐19 during the study period (all the hospitals were the designated to provide inpatient treatment for patients with COVID‐19 by the local government). Thus, those hospitals where in‐hospital treatments for patients with COVID‐19 were provided were classified into an intervention group, whereas those that did not provide were classified into a control group.

### 2.4 Outcome

The outcome of this study was the number of monthly cancer operations performed according to the intervention and control groups. We determined those cases who underwent cancer operations using the Japanese original operation code. The included procedures are summarized in Table S1.

### 2.5 Control variables

The following data were used as control variables and adjusted in our regression analyses: age, sex, Charlson Comorbidity Index (0, 1, and ≥2),^21^ concomitant chemotherapy, cancer recurrence, and population density of hospital location. Age and population density^22^, ^23^ were calculated as the mean age of the intervention and control groups by month. Other covariates were calculated as the monthly proportion by two groups. The variable concerning whether patients had undergone concomitant chemotherapy was included as a control variable to consider the severity of cancer as concomitant chemotherapy is recommended for patients in stages II and III in various cancer treatments.^24^

### 2.6 Data analyses

After descriptive analysis according to the control and intervention groups, an interrupted time series (ITS) analysis was applied to investigate the effects of the different periods of COVID‐19 infection spread on in‐hospital cancer operations.^25^, ^26^, ^27^ The seasonality was adjusted using a Fourier term in addition to the aforementioned control variables in the ITS model.^28^ The ITS analysis enables us to measure two potential changes that could occur due to the exposure via an immediate change in the number of operations performed and a change in the number of operations per month. Our ITS analysis comprised the following three periods: (1) pre‐COVID‐19 period (April 2017 to March 2020), (2) the first wave period (during which inpatient treatment of patients with COVID‐19 was started but not spread too far; April 2020 to November 2020; this period also included the government's emergency declaration [16 April 2020 to 31 May 2020]), and (3) the second wave period (during which infection spread; December 2020 to March 2021). Therefore, for instance, we statistically compared the intervention group with the control group to assess whether a rapid change in cases was observed after the declaration of emergency state by the government. Two types of sensitivity analyses were also performed by changing the time point of the second wave of the infection spread: November 2020 and October 2020.

All analyses were conducted using Stata, version 17.0 (Stata Corp LLC). This study was approved by the ethics committee of Yamagata University (approval no. 2021–115). The requirement for informed consent was waived because of the anonymous nature of the data.

## 3 RESULTS

The characteristics of the study patients stratified by the intervention and control groups are shown in Table 1. Overall, the number of cases was approximately nine times greater in hospitals where in‐hospital treatments for patients with COVID‐19 were provided (control, *n* = 2679; intervention, *n* = 22,395). Moreover, patients who underwent operations for cancer in hospitals that provided inpatient COVID‐19 treatments were more likely to have comorbidity.

**TABLE 1:** Patients' characteristics stratified by intervention and control groups, April 2017 to March 2021

	Control		Intervention	
	*n* = 2679		*n* = 22,395	
Age (mean, standard deviation)	70.8	11.4	69.2	12.1
Women	1140	42.6	9497	42.4
Charlson Comorbidity Index				
0	2003	74.8	11,951	53.4
1	345	12.9	5106	22.9
≥2	331	12.4	5338	23.8
Concomitant chemotherapy	97	3.6	1172	5.2
Whether cancer recurrence	200	7.5	1997	8.9

*Note*: Control: hospitals where in‐hospital treatments for patients with COVID‐19 were not provided (*N* = 8). Intervention: hospitals where in‐hospital treatments for patients with COVID‐19 were provided (*N* = 9). Values are presented as numbers and percentages, unless otherwise noted.

**FIGURE 2:**
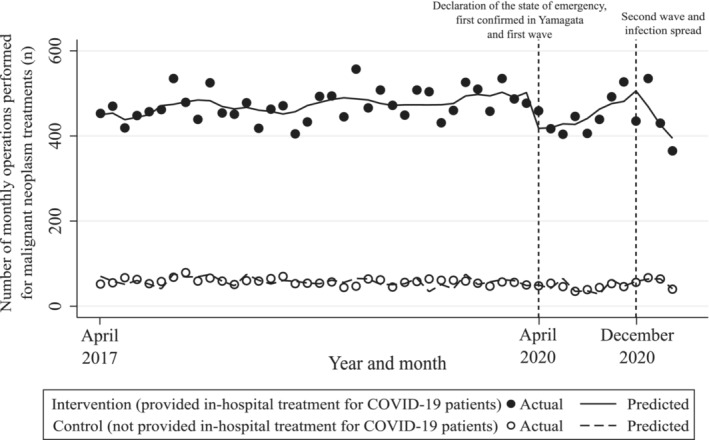
Total number of monthly operations performed for patients with malignant neoplasms, April 2017 to March 2021. The model was adjusted for age, sex, Charlson Comorbidity Index, concomitant chemotherapy, cancer recurrence, population density of hospital location, and seasonality.

Figure 2 and Table 2 show the results of our ITS analysis. First, before March 2020 (the pre‐COVID‐19 period), there was a statistically significant difference in the intercept/trend of the number of cases as well as between the intervention and control groups (coefficient [95% CI] was 381.74 [345.49–418.00] and 1.33 [0.55–2.11], respectively). Second, in April 2020 (the beginning of the first wave period), the difference in the rapid change in the levels between groups was statistically significant; 80.14 operations (95% CI = 39.62–120.67) were reduced immediately after the first wave in the intervention group compared with control group. Third, between December 2020 and March 2021 (the second wave period), the difference in the rapid change in the levels between the groups was not statistically significant; coefficient (95% CI) was −8.96 (−106.12 to 88.20). This result indicated that no significant immediate change in the number of operations performed was observed between the intervention and control groups. However, there was statistical significance for the difference in trends between the groups during this period; 50.67 fewer operations (95% CI = 12.19–89.15) were performed per month in the intervention group compared with control group.

**TABLE 2:** Interrupted time series analysis estimates by different time point, April 2017 to March 2021

Periods	Terms	Coefficient	*p* value	95% confidence interval	
Pre‐COVID‐19 (April 2017 to March 2020)	Difference in level: intervention versus control	**381.74**	**<0.01**	**345.49**	**418.00**
	Difference in trend: intervention versus control	**1.33**	**<0.01**	**0.55**	**2.11**
The first wave period^a^ (not spread too far; April 2020 to November 2020)	Change in level: control	6.44	0.54	−14.58	27.45
	Change in level: intervention	**−73.71**	**<0.01**	**−111.85**	**−35.57**
	Difference in level: intervention versus control	**−80.14**	**<0.01**	**−120.67**	**−39.62**
	Trend: control	−3.65	0.16	−8.74	1.44
	Trend: intervention	7.13	0.19	−3.57	17.82
	Difference in trend: intervention versus control	10.78	0.06	−0.23	21.79
The second wave period (infection spread; December 2020 to March 2021)	Change in level: control	16.15	0.21	−9.23	41.53
	Change in level: intervention	7.19	0.88	−91.01	105.39
	Difference in level: intervention versus control	−8.96	0.86	−106.12	88.20
	Trend: control	9.54	0.17	−4.14	23.22
	Trend: intervention	**−42.47**	**0.03**	**−81.54**	**−3.40**
	Difference in trend: intervention versus control	**−52.01**	**0.01**	**−90.44**	**−13.57**
Difference pre‐COVID‐19 versus the first wave period	Difference in trend: intervention versus control	**12.11**	**0.03**	**1.19**	**23.04**
Difference pre‐COVID‐19 versus the second wave period	Difference in trend: intervention versus control	**−50.67**	**0.01**	**−89.15**	**−12.19**

*Note*: Statistical significance at *p* < 0.05 is indicated in **bold**. Durbin‐Watson statistic = 2.27. The model was adjusted for age, sex, Charlson Comorbidity Index, concomitant chemotherapy, cancer recurrence, population density of hospital location, and seasonality. Control: hospitals where in‐hospital treatments for patients with COVID‐19 were not provided (*N* = 8). Intervention: hospitals where in‐hospital treatments for patients with COVID‐19 were provided (*N* = 9).

^a^ The first wave period included the periods during which inpatient treatment for patients with COVID‐19 was started in Yamagata; this period also included the government's emergency declaration period (16 April 2020, to 31 May 2020).

Table 2 also presents the difference between the intervention and control groups when comparing the differences in trends of the pre‐ and post‐COVID‐19 periods. There was statistical significance for the difference between the groups in the trends of pre‐COVID‐19 and the first and second wave periods; the coefficients (95% CI) were 12.11 (1.19–23.04) and −50.67 (−89.15 to −12.19), respectively. The results indicate that 12.11 more operations (95% CI = 1.19–23.04) were performed per month in the intervention group compared with the control group when comparing the differences in pre‐COVID‐19 and postfirst wave period trends. Meanwhile, 50.67 fewer operations (95% CI = 12.19–89.15) were performed per month in the intervention group compared with the control group when comparing the differences in pre‐COVID‐19 and postsecond wave period trends. Similar results were observed for our sensitivity analyses wherein we altered the time point of the second wave of the infection spread (Table S2).

## 4 DISCUSSION

This study investigated the effects of the different periods of COVID‐19 infection spread on cancer operations using medical claims data in Yamagata. Overall, we confirmed that cancer operations were significantly reduced in hospitals where in‐hospital treatments for patients with COVID‐19 were provided during the COVID‐19 era. However, no significant difference was confirmed in hospitals where in‐hospital treatments for patients with COVID‐19 were not provided.

Two nationwide observational studies from Canada and the United Kingdom reported that the number of treatments for patients with cancer immediately decreased after the first wave of the pandemic (March 2020).^6^, ^7^ Additionally, the number of cancer treatments increased in the UK after the first wave.^7^ Another observational study from 61 countries reported that the lockdown measure was associated with reduced elective cancer surgeries.^29^ These findings were similar to that observed in our study, wherein we found that scheduled operations for patients with cancer immediately decreased in hospitals where in‐hospital treatment for patients with COVID‐19 was provided during the first wave period. The number of cases increased after the first wave until the second wave of the pandemic.

Additionally, we confirmed that the second wave of the pandemic affected cancer operations, although the effect was somewhat different from that in the first wave. Thus, an immediate change in the number of cases was not observed; however, the number of cases dramatically decreased monthly after the second wave in hospitals where in‐hospital treatments for patients with COVID‐19 were provided. The difference between waves is, first, caused by the difference in the number of COVID‐19 cases. The number of COVID‐19 cases rapidly decreased during the first wave of the pandemic; hence, the operations for patients with cancer were assumed to resume. Contrastingly, the number of COVID‐19 cases was relatively larger during the second wave than in the first wave; thus, the number of operations for patients with cancer decreased due to the need to prioritize treatment for patients with COVID‐19. Second, a nationwide state of emergency by the government was only implemented from April 2020 to May 2020 in Yamagata; this state of emergency was a moderate lockdown measure^29^ across all the prefectures of Japan, although the number of COVID‐19 cases in Yamagata was relatively smaller compared with the other prefectures of Japan and other countries.^13^ Thus, the immediate reduction in operations in the first wave in Yamagata might also be due to a nationwide state of emergency by the government rather than the infection spread itself. Therefore, our results underscored the importance of reducing the number of COVID‐19 cases and limiting the spread of COVID‐19 to avoid cancer treatment disruption. Third, there is a difference in patients' reluctance to visit outpatient clinics or hospitals. Patients might refrain from visiting clinics and hospitals amid concerns regarding COVID‐19 transmission, which might consequently influence the number of cancer operations as observed in this study.^30^, ^31^ However, this information was not investigated in the present study; hence, future studies are warranted.

In contrast to the intervention group, neither immediate change in level nor change in trend was statistically significant between the pre‐ and post‐COVID‐19 periods in the control group. The results potentially indicated policymaking for the medical care system of differentiating between hospitals centralized to the treatment of infectious diseases and those centralized to planned treatments, such as cancer operations, in the situations of new infectious diseases epidemic. However, the centralization of cancer treatments may result in inequalities in access to healthcare services. A previous simulation study conducted in Japan reported that centralizing inpatient and outpatient health care services for ischemic heart disease and breast cancer resulted in reduced travel times and improved access inequalities, except those for outpatients with breast cancer.^32^ Therefore, during an infectious disease pandemic, it may be essential to introduce public subsidies to ensure transportation when concentrating medical services for scheduled treatments, such as cancer operations.

In our study, all hospitals that provided in‐hospital treatments for patients with COVID‐19 had a larger number of hospital beds than those that did not provide in‐hospital treatments for patients with COVID‐19. Hospitals with a large number of beds are generally required to provide a high level of multidisciplinary specialist treatment for patients with multiple comorbidities. This is in fact supported by our study findings that cancer patients with several comorbidities were more likely to admit to the hospitals in the intervention group (Table 1). Therefore, rather than transferring these patients with advanced treatment needs to hospitals with fewer beds and facilities, it might be necessary to provide infectious disease and cancer treatments separately in similar hospitals in terms of the size and necessary facilities required to provide advanced treatments. Conversely, careful consideration should be given to cancer treatments that can be provided by general hospitals, as treatments can be provided even without hospitals that provide advanced medical care and may not need to be centralized.

To our knowledge, this is the first study to investigate the impact of the different periods of COVID‐19 infection spread on cancer operations with intervention and control groups. Hence, we could add to the evidence that the COVID‐19 pandemic influenced the disruption of cancer operations. Moreover, in Yamagata prefecture, it was possible to monitor the changes in the number of COVID‐19 cases over time and periods when the number of infected people was low. Therefore, this research was the so‐called “natural experimental design” that allowed us to investigate the effects of the different periods of COVID‐19 infection spread on cancer operations.

Nevertheless, the present study had several limitations. First, the generalizability of our study findings may be limited to areas with large population density where the number of infected people rapidly increases. In Japan, COVID‐19 cases were more likely to be confirmed in prefectures with larger population densities than in those with smaller population densities. Additionally, we only focused on those inpatients who underwent cancer operations. Therefore, our findings might not be generalized to nonoperation cancer treatments. Amid the pandemic, there may be an increasing number of patients who underwent nonoperation treatments, depending on cancer type and location.^33^, ^34^ However, the impact of such alternative treatments on the operation is unclear and was therefore excluded from this study.^33^, ^34^ Second, our findings might not be generalizable depending on the cancer site or operation type. Therefore, future studies with larger sample size data with separate cancer sites and operation types are warranted. Third, the DPC data used in this study are routinely collected every year according to the fiscal year (i.e. April–March). Therefore, the available data at the time the study was conducted were till March 2021, and exploring the impact of the COVID‐19 pandemic on cancer operations was not possible after the third wave (i.e. March 2021 to June 2021) when the number of confirmed COVID‐19 cases showed a greater increase than that in the previous waves. Hence, future studies with longer time periods are warranted. Fourth, our data might be affected by confounders, such as bed occupancy for patients with COVID‐19 infection, although we used a “natural experiment design” using the induced exogenous variation by the COVID‐19 infection spread. Fifth, we did not consider the timing of the designation of hospitals to provide inpatient treatment for patients with COVID‐19. Three out of the nine hospitals in the intervention group did not provide inpatient treatment for patients with COVID‐19 during the first wave, and thus, our estimates for the first wave might have included biased results.

## 5 CONCLUSIONS

We investigated the effects of the different periods of COVID‐19 infection spread on scheduled operations for patients with cancer using routinely collected medical claims data from multiple hospitals in Yamagata. Our findings suggest that a statement of emergency by the government and the COVID‐19 infection spread are both associated with the number of cancer operations performed in Yamagata prefecture during the COVID‐19 pandemic.

## AUTHOR CONTRIBUTIONS

Ikeda, Kashiwagura, and Murakami had full access to all of the data in the study and takes responsibility for the integrity of the data and the accuracy of the data analysis. Concept and design: Kashiwagura, Motoi, Cooray, Osaka, Murakami, Ikeda. Acquisition, analysis, or interpretation of data: Kashiwagura, Motoi, Cooray, Osaka, Murakami, Ikeda. Drafting of the manuscript: Kashiwagura. Critical revision of the manuscript for important intellectual content: All authors. Statistical analysis: Kashiwagura, Cooray, Ikeda. Obtained funding: Murakami, Ikeda. Supervision: Motoi, Osaka.

## FUNDING INFORMATION

This study was conducted as part of the “Policy Research on the Realization of the Regional Medical Care Plan and the Allocation of Medical Doctors” commissioned by Yamagata Prefecture, Japan (MM). This work was supported by the Japan Society for the Promotion of Science (JSPS) KAKENHI Grant Number (19K19818, 22K17648, TI) and the Institute for Health Economics and Policy Foundation (TI). No sponsor had any role in the design or conduct of the study; collection, management, analysis, or interpretation of the data; preparation, review, or approval of the manuscript; or the decision to submit the manuscript for publication. The authors have no financial competing interests.

## CONFLICT OF INTEREST

None of the authors of this manuscript have any conflicts of interests to report.

## Supporting information


Table S1–S2

## Data Availability

Our data are not available for publication because we are restricted from making the minimal dataset publicly available due to data protection regulations concerning the secured management of DPC data. Our data is obtained from hospitals by this contractual compliance. The data can be accessed only by those who meet the criteria for these confidential data. Requests to access the data should be submitted to Professor Masayasu Murakami, Department of Health Policy Science, Graduate School of Medical Science, Yamagata University, Yamagata, Japan. E‐mail: mmurakami@med.id.yamagata‐u.ac.jp
