# Supplementary material for: Effects of the different periods and magnitude of COVID‐19 infection spread on cancer operations: Interrupted time series analysis of medical claims data
Source: Cancer Med. 2022 Sep 20;12(4):4707–14. doi: 10.1002/cam4.5259 (PMC9538599; doi:10.1002/cam4.5259)
Supplement: Supplementary file 1 — Table S1–S2 [file CAM4-12-4707-s001.docx]

Supplementary Materials

Supplementary Table 1. Cancer operations in this study.

| Procedures |
| --- |
| Endoscopic submucosal resection of gastric and duodenal polyps |
| Local resection of the stomach |
| Gastrectomy surgery for malignant tumor |
| Total gastrectomy surgery for malignant tumor |
| Proximal gastrectomy |
| Laparoscopic local resection of the stomach |
| Laparoscopic gastrectomy |
| Laparoscopic total gastrectomy |
| Laparoscopic proximal gastrectomy |
| Lip surgery for malignant tumor |
| Tongue surgery for malignant tumor |
| Oral floor surgery for malignant tumor |
| Epipharynx surgery for malignant tumor |
| Pharynx surgery for malignant tumor |
| Hypopharynx surgery for malignant tumor |
| Endoscopic pharyngeal head surgery for malignant tumor |
| Laryngeal surgery for malignant tumor |
| Endoscopic laryngeal surgery for malignant tumor |
| Oral, jaw, and face surgery for malignant tumor |
| Extraction of parapharyngeal space surgery for malignant tumor |
| Maxilla surgery for malignant tumor |
| Mandibular surgery for malignant tumor |
| Submandibular Gland surgery for malignant tumor |
| Endoscopic nasal and paranasal surgery |
| Nasal and paranasal surgery for malignant tumor |
| Buccal region surgery for malignant tumor |
| Buccal mucosa surgery for malignant tumor |
| Middle ear surgery for malignant tumor |
| External auditory canal surgery for malignant tumor |
| Parotid gland surgery for malignant tumor |
| Thyroid surgery for malignant tumor |
| Endoscopic thyroid surgery for malignant tumor |
| Cervical surgery for malignant tumor |
| Parathyroid surgery for malignant tumor |
| Hepatectomy |
| Laparoscopic hepatectomy |
| Eyelid conjunctival surgery for malignant tumor |
| Extraction of conjunctival tumor |
| Extensive skull base reconstruction and reconstruction |
| Extraction of intracranial tumor |
| Scalp and skull surgery for malignant tumor |
| Endoscopic nasal extraction of skull base tumor |
| Endoscopic extraction of esophageal submucosal tumor |
| Esophageal surgery for malignant tumor |
| Esophageal reconstruction after ablative surgery |
| Thoracoscopic esophageal surgery for malignant tumor |
| Extraction of bronchial tumor |
| Extraction of chest wall surgery for malignant tumor |
| Lung surgery for malignant tumor |
| Lung resection |
| Thoracoscopic lung surgery for malignant tumor |
| Thoracoscopic lung surgery for malignant tumor |
| Thoracoscopic mediastinum surgery for malignant tumor |
| Mediastinum surgery for malignant tumor |
| Pericardial surgery for tumor |
| Breast surgery for malignant tumor |
| Microdiscectomy |
| Lumpectomy |
| Mastectomy |
| Renal (ureteral) surgery for malignant tumor |
| Laparoscopic renal (ureteral) surgery for malignant tumor |
| Laparoscopic renal surgery for malignant tumor |
| Adrenal surgery for malignant tumor |
| Laparoscopic adrenal surgery for malignant tumor |
| Retroperitoneal surgery for malignant tumor |
| Extraction of omentum, mesentery and retroperitoneal tumors |
| Extraction of Abdominal Wall tumor |
| Pancreatectomy |
| Distal pancreatectomy surgery for malignant tumor |
| Laparoscopic distal pancreatectomy surgery for tumor |
| Pancreaticoduodenectomy |
| Pancreas head surgery for tumor |
| Total pancreatectomy |
| Hepatic cholangitis surgery for malignant tumor |
| Bile duct surgery for malignant tumor |
| Gallbladder surgery for malignant tumor |
| Enterectomy |
| Laparoscopic enterectomy |
| Endoscopic submucosal resection of colonic polyps |
| Endoscopic submucosal dissection of early colonic malignant tumor |
| Laparoscopic enterectomy for malignant tumor |
| Colectomy |
| Laparoscopic enterectomy |
| Extraction of rectum tumor |
| Rectal resection |
| Laparoscopic rectal resection |
| Anus surgery for malignant tumor |
| Extraction of urethral surgery for malignant tumor |
| Laparoscopic ureteral surgery for tumor |
| Extraction of Urachal |
| Laparoscopic bladder surgery for malignant tumor |
| Bladder surgery for malignant tumor |
| Penile surgery for malignant tumor |
| Testicular surgery for malignant tumor |
| Orchidectomy |
| Prostate surgery for malignant tumor |
| Laparoscopic prostate surgery for malignant tumor |
| Uterine cervix resection |
| Vaginal wall surgery for malignant tumor |
| Uterine surgery for malignant tumor |
| Laparoscopic uterine surgery for malignant tumor |
| Uterine annex surgery for malignant tumor |
| Female genitalia surgery for malignant tumor |
| Total hysterectomy |
| Laparoscopic vaginal hysterectomy |
| Skin surgery for malignant tumor |
| Forequarter amputation |
| Bone surgery for malignant tumor |
| Pelvic resection |
| Total pelvic exenteration |
| Upper/lower limb disarticulation |
| Limb amputation |
| Spine and pelvic surgery for malignant tumor |
| Hematopoietic stem cell transplantation |

Supplementary Table 2. Sensitivity analyses by altering the time point of the second wave infection spread: Interrupted time-series analysis estimates by different points, April 2017 to March 2021.

| Periods | Terms | Coefficient | *P* value | 95% confidence interval | |
| --- | --- | --- | --- | --- | --- |
| **The time point of the second wave of the infection spread = November 2020** | | | | | |
| Pre-COVID-19 (April 2017 to March 2020) | Difference in level: intervention vs. control | **383.71** | **<0.01** | **346.77** | **420.65** |
|  | Difference in trend: intervention vs. control | **1.34** | **<0.01** | **0.56** | **2.11** |
| The first wave period* (not spread too far; **April 2020 to November 2020**) | Change in level: control | 4.56 | 0.66 | −16.26 | 25.37 |
|  | Change in level: intervention | **−63.06** | **<0.01** | **−96.95** | **−29.17** |
|  | Difference in level: intervention vs. control | **−67.61** | **<0.01** | **−104.24** | **−30.99** |
|  | Trend: control | −3.32 | 0.26 | −9.15 | 2.51 |
|  | Trend: intervention | 2.34 | 0.61 | −6.88 | 11.57 |
|  | Difference in trend: intervention vs. control | 5.66 | 0.25 | −4.13 | 15.45 |
| The second wave period (**November 2020 to March 2021**) | Change in level: control | −1.21 | 0.93 | −29.63 | 27.21 |
|  | Change in level: intervention | 61.14 | 0.15 | −23.14 | 145.42 |
|  | Difference in level: intervention vs. control | 62.35 | 0.15 | −21.91 | 146.62 |
|  | Trend: control | **12.09** | **0.03** | **0.97** | **23.22** |
|  | Trend: intervention | **−34.25** | **0.01** | **−57.84** | **−10.66** |
|  | Difference in trend: intervention vs. control | **−46.34** | **<0.01** | **−71.28** | **−21.41** |
| Difference pre-COVID-19 vs. the first wave period | Difference in trend: intervention vs. control | 7.00 | 0.16 | −2.73 | 16.73 |
| Difference pre-COVID-19 vs. the second wave period | Difference in trend: intervention vs. control | **−45.01** | **<0.01** | **−70.01** | **−20.00** |
| **The time point of the second wave of the infection spread = October 2010** | | | | | |
| Pre-COVID-19 (April 2017 to March 2020) | Difference in level: intervention vs. control | **386.81** | **<0.01** | **348.44** | **425.18** |
|  | Difference in trend: intervention vs. control | **1.34** | **<0.01** | **0.58** | **2.10** |
| The first wave period* (not spread too far; **April 2020 to October 2020**) | Change in level: control | 3.64 | 0.73 | −17.26 | 24.53 |
|  | Change in level: intervention | **−50.54** | **<0.01** | **−79.41** | **−21.66** |
|  | Difference in level: intervention vs. control | **−54.17** | **<0.01** | **−88.10** | **−20.25** |
|  | Trend: control | −2.71 | 0.45 | −9.86 | 4.43 |
|  | Trend: intervention | −4.99 | 0.16 | −12.01 | 2.04 |
|  | Difference in trend: intervention vs. control | −2.27 | 0.56 | −10.09 | 5.54 |
| The second wave period (**October 2020 to March 2021**) | Change in level: control | −9.86 | 0.56 | −43.57 | 23.84 |
|  | Change in level: intervention | **98.01** | **<0.01** | **51.72** | **144.30** |
|  | Difference in level: intervention vs. control | **107.87** | **<0.01** | **59.71** | **156.04** |
|  | Trend: control | 9.75 | 0.05 | −0.09 | 19.58 |
|  | Trend: intervention | **−18.99** | **0.03** | **−35.59** | **−2.39** |
|  | Difference in trend: intervention vs. control | **−28.73** | **<0.01** | **−45.85** | **−11.61** |
| Difference pre-COVID-19 vs. the first wave period | Difference in trend: intervention vs. control | −0.94 | 0.81 | −8.67 | 6.80 |
| Difference pre-COVID-19 vs. the second wave period | Difference in trend: intervention vs. control | **−27.40** | **<0.01** | **−44.60** | **−10.19** |

Statistical significance at P < 0.05 is indicated in **bold**.

The model was adjusted for age, sex, Charlson Comorbidity Index, concomitant chemotherapy, cancer recurrence, population density of hospital location and seasonality.

Control: hospitals where in-hospital treatments for patients with COVID-19 were not provided (N = 8).

Intervention: hospitals where in-hospital treatments for patients with COVID-19 were provided (N = 9).

* The first wave period included the periods during which inpatient treatment for patients with COVID-19 was started in Yamagata; this period also included the government’s emergency declaration period (April 16, 2020, to May 31, 2020).
